# Production of two SARS-CoV-2 neutralizing antibodies with different potencies in *Nicotiana benthamiana*

**DOI:** 10.3389/fpls.2022.956741

**Published:** 2022-09-05

**Authors:** Rachele Frigerio, Carla Marusic, Maria Elena Villani, Chiara Lico, Cristina Capodicasa, Emanuele Andreano, Ida Paciello, Rino Rappuoli, Anna Maria Salzano, Andrea Scaloni, Selene Baschieri, Marcello Donini

**Affiliations:** ^1^Laboratory of Biotechnology, ENEA Research Center Casaccia, Rome, Italy; ^2^Monoclonal Antibody Discovery (MAD) Lab, Fondazione Toscana Life Sciences, Siena, Italy; ^3^Department of Biotechnology, Chemistry and Pharmacy, University of Siena, Siena, Italy; ^4^Proteomics, Metabolomics and Mass Spectrometry Laboratory, ISPAAM, National Research Council, Portici, Italy

**Keywords:** molecular farming, COVID-19, transient expression, proteolytic degradation, monoclonal antibodies (mAbs)

## Abstract

Monoclonal antibodies are considered to be highly effective therapeutic tools for the treatment of mild to moderate COVID-19 patients. In the present work, we describe the production of two SARS-CoV-2 human IgG1 monoclonal antibodies recognizing the spike protein receptor-binding domain (RBD) and endowed with neutralizing activity (nAbs) in plants. The first one, mAbJ08-MUT, was previously isolated from a COVID-19 convalescent patient and Fc-engineered to prolong the half-life and reduce the risk of antibody-dependent enhancement. This nAb produced in mammalian cells, delivered in a single intramuscular administration during a Phase I clinical study, was shown to (i) be safe and effectively protect against major variants of concern, and (ii) have some neutralizing activity against the recently emerged omicron variant in a cytopathic-effect-based microneutralization assay (100% inhibitory concentration, IC_100_ of 15 μg/mL). The second antibody, mAb675, previously isolated from a vaccinated individual, showed an intermediate neutralization activity against SARS-CoV-2 variants. Different accumulation levels of mAbJ08-MUT and mAb675 were observed after transient agroinfiltration in *Nicotiana benthamiana* plants knocked-out for xylosil and fucosil transferases, leading to yields of ~35 and 150 mg/kg of fresh leaf mass, respectively. After purification, as a result of the proteolytic events affecting the hinge-CH2 region, a higher degradation of mAb675 was observed, compared to mAbJ08-MUT (~18% vs. ~1%, respectively). Both nAbs showed a human-like glycosylation profile, and were able to specifically bind to RBD and compete with angiotensin-converting enzyme 2 binding *in vitro*. SARS-CoV-2 neutralization assay against the original virus isolated in Wuhan demonstrated the high neutralization potency of the plant-produced mAbJ08-MUT, with levels (IC_100_ < 17 ng/mL) comparable to those of the cognate antibody produced in a Chinese hamster ovary cell line; conversely, mAb675 exhibited a medium neutralization potency (IC_100_ ~ 200 ng/mL). All these data confirm that plant expression platforms may represent a convenient and rapid production system of potent nAbs to be used both in therapy and diagnostics in pandemic emergencies.

## Introduction

Given the urgency of containing the spread of SARS-CoV-2, recombinant antibodies having virus neutralization properties (neutralizing antibodies, nAbs), interacting with the spike protein (S-protein) and preventing the pathogen's binding to the human angiotensin-converting enzyme 2 (hACE2) receptor present on host cells, have been produced in mammalian cell factories and are currently in clinical trials (Li et al., 2022). Some of them have been approved with a special procedure for emergency use, such as Regeneron's casirivimab (REGN10933) and imdevimab (REGN10987) and Eli Lilly and Company's bamlanivimab and etesevimab combinations (Gottlieb et al., 2021; Weinreich et al., 2021). In this context, plant molecular farming and transient transformation techniques offer a rapid and low-cost platform for the production of nAbs. They can be considered as a valid alternative to mammalian cell cultures in emergency situations mainly due to the rapidity of the process and the fact that it is not necessary to develop stably transformed cell lines. Moreover, the scalability of transient expression systems can be easily adapted by just increasing the cultivation area with low investments (Capell et al., 2020; Tusé et al., 2020). On this basis, the potential of plant expression systems for the production of human anti-SARS-CoV-2 monoclonal antibodies (mAbs) to be used for therapeutic and diagnostic applications has been explored in several studies. A first example is represented by the transient expression of two human mAbs against SARS-CoV-2 (B38 and H4) using viral vectors in *Nicotiana benthamiana* (Shanmugaraj et al., 2020). In the following study, a human mAb (CR3022) specific for SARS coronavirus but also able to bind to the receptor-binding domain (RBD) of SARS-CoV-2 was efficiently produced in *N. benthamiana* but was not found to be effective in *in vitro* neutralization tests (Rattanapisit et al., 2020). Through a third approach, it was demonstrated that plant biofactories can offer rapid and easily adaptable solutions for local manufacturing of bioreagents to be used in serological and antigen detection kits. In particular, it was shown that recombinant antibodies against SARS-CoV-2, engineered as single-chain fragments fused to a human gamma chain constant region (Fc), can be rapidly and efficiently produced by transient expression using viral vectors in *N. benthamiana* (Diego-Martin et al., 2020).

Despite the fact that human mAbs are considered as efficacious biopharmaceuticals to tackle pandemics, as demonstrated by the approval for human use of several SARS-CoV-2 neutralizing antibodies, the development of novel nAbs with high potency still remains a priority, considering the continuous and rapid occurrence of new viral variants. It is also important to identify production systems for these biopharmaceuticals allowing rapid scale-up and reduction of costs. Neutralizing antibodies against RBD can be classified into four groups depending on their specific binding site to the S-protein and their ability to block interaction with ACE2. The most represented ones are those belonging to Class 1 that are able to block ACE2 and bind only to RBD in the “Up” conformation (Barnes et al., 2020). The mAb675, isolated from a vaccinated patient, was described as an “unclassified” antibody, as it was not possible to verify its binding modalities to RBD, although the heavy chain encoded by the *VH3-53* gene and the short complementarity-determining region (CDR) H3 loop (nine residues) were predicted as belonging to Class 1 nAbs (Barnes et al., 2020). The mAbJ08, which was isolated from a COVID-19 convalescent patient, targets an epitope between Class 1 and 2, as it is able to recognize the RBD in both “Up” and “Down” conformations (Torres et al., 2022). Different from mAb675, the heavy chain (HC) of mAbJ08 is encoded by the *VH1-69* gene and has a longer CDR H3 loop (16 residues). Interestingly, this particular germline gene is predominant in SARS-CoV-2 nAbs and has often been found in broadly effective nAbs. It must be noted that mAbJ08 has a short CDR-L3 (five residues), which is also a common feature of many broadly effective nAbs (DeKosky et al., 2015). This nAb was optimized (mAbJ08-MUT) to reduce the risk of antibody-dependent enhancement (ADE) (Chen et al., 2019), and five different point mutations were introduced in the Fc region to increase the half-life and reduce antibody-dependent functions (Andreano et al., 2021). Both antibodies produced in mammalian cells proved to maintain their neutralizing capacity against the different SARS-CoV-2 variants circulating at the beginning of 2021 with mAbJ08-MUT, in particular, confirming the extremely high neutralization potency (IC_100_ lower than 10 ng/mL) against both the SARS-CoV-2 virus strains isolated in Wuhan and variants of concern (Alpha B.1.1.7, Beta B.1.351, Gamma P.1, and Delta B.1.617.2), and also exhibiting some neutralization activity against B.1.1.529 variant (Omicron) (Torres et al., 2022). A recent Phase I clinical trial evidenced that a single intramuscular administration of this nAb (48 mg) is sufficient to induce sera neutralizing titers higher than those found in COVID-19 convalescent and vaccinated individuals (Lanini et al., 2022).

In this work, we produced the two previously described antibodies (mAb675 and mAbJ08-MUT) by transient expression in *N. benthamiana* plants modified for the glycosylation profile (Jansing et al., 2019). In particular, we report the effect of different cultivation conditions (hydroponics on inorganic substrate or soil) on antibody accumulation levels and the possible effect of the Fc mutations on the accumulation and degradation of mAbJ08, exploring also the possible influence of light chain (LC) expression yields on the overall accumulation levels of mAbs.

## Materials and methods

### Recombinant antibody constructs

The HC and LC encoding genes of anti-SARS-CoV-2 mAbs were synthetically constructed and codon-optimized for expression in *N. benthamiana* using the OptimumGene^TM^ algorithm (GenScript, Piscataway NJ, USA). The sequences were cloned into the plant expression binary vector pBI-Ω (Marusic et al., 2007) using the BamHI/EcoRI restriction sites, yielding plasmids pBI-ΩJ08-MUT-HC, pBI-ΩJ08-MUT-LC, pBI-Ω675-HC, and pBI-Ω675-LC (Supplementary Figure S1). All sequences encode the PR1 signal peptide sequence from tobacco, UniProtKB-P08299 (PR1A_TOBAC). The pBI-Ωp19 bearing the *p19* silencing suppressor gene from the artichoke mottled crinkle virus (AMCV) was also used (Marusic et al., 2007).

### Plant growth and transient expression

*Nicotiana benthamiana* plants were either grown in pots with soil or on rockwool culture substrate (cultilene 25/150) (Cultilene Grodan, Rijen the Netherlands) using a hydroponic system with an Ebb and flow type irrigation with a nutrient solution diluted 1:200 in water (Hydro Grow type nutrient solution, Growth Technology Ltd, Taunton, UK), one flood every 48 h (6.0–6.5 pH values and electric conductivity values of 1–1.5 mS/cm). Both in soil and on rockwool substrate, germination was performed at a temperature of 24°C, 80% humidity, and with light at an intensity of 80 μmol m^2^s^−1^. Seeds were either sowed in soil or in rockwool trays (Cultilene 2.5 × 2.5 cm) and after 10 days from sowing, the seedlings were either transferred to pots or to larger rockwool trays (Cultilene 7.5 × 7.5 cm), and growth was obtained at 24°C under LED lamps (Valoya AP673L, Helsinki, Finland) at an intensity of 140 μmol m^2^s^−1^, with a 16 h light and 8 h dark cycle. The plant-codon-optimized sequences encoding for the HC and LC of mAb675 (Wang et al., 2021) and mAbJ08-MUT (Andreano et al., 2021) were cloned into the pBI-Ω plant expression vector and used to transform *Agrobacterium tumefaciens* (LBA 4404). The expression of each antibody was performed in fucosyl and xylosyl transferase knock-out (FX-KO) *N. benthamiana* plants optimized for the glycosylation profile by genome editing (Jansing et al., 2019). Transient expression in plant leaves was performed either by using a syringe or vacuum agroinfiltration. *A. tumefaciens* clones harboring mAbJ08-MUT-HC and LC, mAb675-HC and LC, and p19 constructs were grown separately. Bacteria were pelleted by centrifugation at 4,000 ×*g* and resuspended in infiltration buffer (10 mM MES, 10 mM MgCl_2_, pH 5.8). Agrobacterium suspensions harboring the different vectors were mixed together (mAb-HC, mAb-LC, and p19 at 1:1:1 ratio) reaching a final OD_600_ of 0.5 for each construct (Lombardi et al., 2009). To determine the accumulation of mAbsJ08-MUT and mAb675 at different time points, as well as of the chimeric mAbs obtained by swapping HC and LC between the two antibodies, 6-week-old *N. benthamiana* plants (at the 6–7 leaf stage) were syringe infiltrated, and leaf sampling was performed at set time points (2–9 days post-infiltration). Leaves from three individual plants were collected, and antibody accumulation yields were assayed by comparing vacuum-agroinfiltrated plants grown in soil or in a hydroponic system. To this aim, plants at the 6-7 leaf stage were infiltrated by completely submerging each plant in the *Agrobacterium*-containing solution inside a vacuum chamber. The vacuum was applied reaching ~1.3 kPa, and then quickly released. Plants were then grown for another 9 days post-infiltration in the same conditions used before (light intensity of 140 μmol m^2^s^−1^, with a 16-h light and 8-h dark cycle at 24°C). For antibody purification, 40-g batches of agroinfiltrated leaves were collected, frozen in liquid N_2_, and stored at - 80°C before use.

### ELISA, SDS-PAGE, and western blot analysis

To quantify mAb expression, crude leaf extracts from agroinfiltrated leaves were analyzed by Double-Antibody-Sandwich (DAS)-ELISA. Briefly, leaf tissue (100 mg) was ground in liquid N_2_ and homogenized in 500 μL of phosphate-buffered saline (PBS, pH 7.2) containing protease inhibitors (Complete^TM^, Roche, Basel, Switzerland). After centrifugation at 20,000 ×*g* at 4°C for 30 min, the supernatant was recovered and quantified for total soluble proteins (TSPs) using the Bradford colorimetric assay (Bio-Rad, Hercules, CA, USA). The capture antibody (anti-human γ chain, I6010, Sigma-Aldrich, St. Louis, MO, USA), at a concentration of 2 μg L^−1^ in PBS (200 ng per well), was coated directly onto Nunc-Immuno Maxisorp wells and incubated overnight at 4°C. Plates were blocked with 4% (m/v) milk in PBS at 37°C for 2 h; after washing, different dilutions of the leaf extracts, normalized for TSP concentration, were added to the wells (100 μL) and incubated at 37°C for 2 h. The anti-human γ chain HRP-conjugated antibody (A18853, Thermo Fischer Scientific, Waltham, MA, USA) was added at a concentration of 0.2 mg L^−1^ in PBS containing 2% (m/v) milk, and incubated at 37°C for 1 h. Enzymatic activity was measured after 30 min at 405 nm using a microtiter plate reader (TECAN-Sunrise, Mannedorf, Switzerland) and 2,2-azino-di-3-ethylbenzothiazoline sulfonate (ABTS, KPL. Seracare Life Sciences, Milford, MA, USA) as substrate. As a positive control, the purified mAb675 and mAbJ08-MUT from plants were used in ELISA at different dilutions (ranging from 1 to 100 ng). The binding of plant extracts to RBD was assayed by ELISA coating plates with 150 ng/well of recombinant SARS-CoV-2 Spike Protein RBD mFc Tag containing a mouse Fc portion (RP-87700 Thermo Fischer Scientific, Waltham, MA, USA). After blocking with 4% (m/v) milk in PBS at 37°C for 2 h, plant extracts at different dilutions (1:25 to 1:100) were added to wells and incubated at room temperature (20–22°C) for 2 h. The anti-human γ chain HRP-conjugated antibody (8419, Sigma-Aldrich, St. Louis, MO, USA) at a dilution of 1:5,000 was used as a secondary antibody. Enzymatic activity was measured as described above.

A competitive ELISA (SARS-CoV-2 Neutralizing Antibody ELISA Kit, Thermo Fisher Scientific, Waltham, MA, USA) was used to assay the capacity of plant-produced nAbs to compete for binding to ACE2 with RBD, following the manufacturer's instructions. Briefly, antibodies (50 and 100 ng/mL) and serum samples (1:50 dilution) from a vaccinated individual (two doses of Pfizer–BioNTech vaccine) and pre-vaccine serum from the same individual (C-) were incubated in RBD-coated wells (100 μL) for 30 min at room temperature. The wells were washed three times, and 100 μL of biotinylated ACE2 was added; the resulting mixtures were incubated for 30 min at room temperature. The plate was washed again, and then 100 μL of the streptavidin-HRP conjugate was added and left to incubate for 15 min at room temperature. After further washing, 100 μL of substrate solution was added and left to incubate for another 15 min, after which the enzymatic reaction was stopped. Absorbance was measured at 450 nm on a microtiter plate reader (TECAN-Sunrise, Mannedorf, Switzerland).

Plant extracts were analyzed by Western blotting, and separation was performed on 10 or 12% SDS-PAGE acrylamide gels. The proteins were electrotransferred onto a PVDF membrane (Trans-Blot Turbo Mini 0.2 μm PVDF Transfer Packs, Bio-Rad, Hercules, CA, USA) using a Semi-Dry Transfer Unit (Trans-Blot^®^ Turbo^™^ Transfer System 1704150, Bio-Rad, Hercules, CA, USA). Membranes were blocked with PBS containing 4% (m/v) milk overnight, before adding anti-human γ chain HRP-conjugated (8419, Sigma-Aldrich, St. Louis, MO, USA) or anti-human γ chain HRP-conjugated (A18853, Thermo Fisher Scientific Waltham, MA, USA) antibodies, at a dilution of 0.2 mg L^−1^ in PBS containing 2% (m/v) milk. Proteins were detected by enhanced chemiluminescence (ECL™ Prime Western Blotting System, Merck, Darmstadt, Germany). As a control, commercial Rituximab (mAbRTX) was used (hcd20-mab1, Invivogen, San Diego, CA, USA). To measure the binding of mAb675 and mAbJ08-MUT to recombinant RBD in Western blot, 25 ng of the commercial RBD fusion protein (~ 51 kDa) produced in HEK293 cells (SARS-CoV-2 Spike Protein RBD mFc Tag Recombinant- RP-87700 Thermo Fischer Scientific) were separated on non-reducing 12% SDS-PAGE, and assayed with both antibodies at a dilution of 200 ng/mL followed by treatment with anti-human γ chain HRP-conjugated antibody (8419, Sigma-Aldrich, St. Louis, MO, USA). As a control, a rabbit anti-RBD polyclonal antibody (SARS Coronavirus Spike Protein antibody-PA5-81795, Thermo Fisher Scientific, Waltham, MA, USA) at 1:5,000 dilution was used, followed by treatment with HRP-conjugated anti-rabbit antibody (0545, Sigma-Aldrich, St. Louis, MO, USA) at a dilution of 1:10,000 in PBS containing 2% (m/v) milk. Proteins were detected by enhanced chemiluminescence (ECL^™^ Prime Western Blotting System, Merck, Darmstadt, Germany).

### Protein-A affinity chromatography and size-exclusion chromatography

Antibodies were extracted from agroinfiltrated leaves and purified by protein-A affinity chromatography. Batches of 40 g of leaves were ground in liquid N_2_ to a fine powder and homogenized with an Ultra-Turrax homogenizer T25 (IKA, Staufen, Germany) in 80 mL of PBS (pH 7.2), 0.2% v/v Tween, and containing protease inhibitor (Complete^TM^ Roche, Basel, Switzerland) (2 mL/g fresh tissue). The slurry was filtered through Miracloth having a pore size of 22–25 μm (Sigma-Aldrich, St. Louis, MO, USA) and clarified by double centrifugation at 8,000 ×*g* for 20 min at 4°C. The supernatant was loaded onto a protein-A affinity column (1 mL HiTrap^™^ Protein-A FF, GE Healthcare, Chicago, IL, USA) at a flow rate of 1 mL/min. The column was washed with 10 column volumes of PBS, and each antibody was eluted with 200 mM Tris-HCl and 100 mM glycine (pH 3.0). Eluted fractions (0.5 mL each) were neutralized to about pH 7.0 with 100 μL of 1M Tris-HCl (pH 9.5) and analyzed by SDS-PAGE, followed by Coomassie blue staining. As a control, commercial Rituximab (mAbRTX) was used (hcd20-mab1, Invivogen, San Diego, CA, USA). Eluted fractions were pooled, passed through a PD10 column (GE Healthcare, Chicago, IL, USA) in PBS, and concentrated in Centriprep YM-3 (Millipore, Burlington, MA, USA) filtration devices. Antibody concentration was determined by measuring the corresponding absorbance at 280 nm, and purity was evaluated by SDS-PAGE, followed by Coomassie blue staining. Purified mAbs were analyzed by size-exclusion chromatography on a Superdex^TM^ 75 10/300 GL column with a bed volume of 24 mL (GE Healthcare, Chicago, IL, USA) linked to an ÄKTA FPLC P920 instrument (GE Healthcare, Chicago, IL, USA), which was eluted with PBS at a flow rate of 0.3 mL/min at 20°C, as described before (Lombardi et al., 2010). Protein absorbance expressed as absorption units (mAU) was measured at 280 nm. Column calibration was performed using dedicated calibration kits (Low and High Molecular Weight, GE Healthcare, Chicago, IL, USA), according to the manufacturer's instructions. Peaks representing different protein fractions were collected using a FRAC-920 fraction collector (GE Healthcare, Chicago, IL, USA).

### Proteomic and glycosylation analyses

Purified antibodies were separated on a non-reducing 10% SDS-PAGE; the gel was stained with colloidal Coomassie, and the protein bands were excised, triturated, *in-gel* reduced and S-alkylated with iodoacetamide, and finally digested with trypsin (Roche, Basel, Switzerland) (Salzano et al., 2013). An aliquot of the peptide mixture was directly analyzed by MALDI-TOF-MS on an Ultraflextreme instrument (Bruker Daltonics, Billerica, MA, USA) operating in reflectron mode (acquisition range *m/z* 400-6,200, pulsed ion extraction 100 ns, and laser frequency 1,000 Hz). 2,5-Dihydroxy-benzoic acid (10 mg/mL in 50% v/v acetonitrile and 0.1% v/v trifluoroacetic acid) was used as a matrix and mixed in a 1:1 ratio to the sample before loading on the instrument target. Mass spectra were calibrated externally using Peptide Calibration Standard II (Bruker Daltonics, Billerica, MA, USA) and elaborated using the FlexAnalysis software (Bruker Daltonics, Billerica, MA, USA).

Peptides were extracted from the gel particles using 5% formic acid/acetonitrile (1:1 v/v), and digest solutions were desalted by using μZipTipC18 pipette tips (Millipore, Burlington, MA, USA). Protein digests were finally analyzed by nanoLC-ESI-Q-Orbitrap MS/MS using an LTQ XL Q-ExactivePlus mass spectrometer equipped with a Nanoflex ion source and connected to an UltiMate 3000 HPLC RSLC nano system-Dionex (Thermo Fisher Scientific, Waltham, MA, USA). Peptides were separated on an Acclaim PepMap RSLC C18 column (150 mm length × 75 μm internal diameter, 2-μm particle size, and 100-Å pore size) (Thermo Fischer Scientific, Waltham, MA, USA) as previously reported (Lonoce et al., 2019). Full mass spectra were acquired in the range *m/z* 375–1,500, with a nominal resolution of 70,000 using a data-dependent scanning procedure over the eight most abundant ions, using 20-s dynamic exclusion. Mass isolation window and collision energy were set to *m/z* 1.2 and 28%, respectively.

NanoLC-ESI-Q-Orbitrap MS/MS data were searched with Byonic^™^ software (v.2.6.46) (Protein Metrics, Cupertino, USA) against a database containing the sequence of the recombinant mAb675 and mAbJ08-MUT and common contaminants. Searching parameters were trypsin as cleavage specificity, allowing also semi-specific cuts and two missed cleavages as the maximum value, Cys carbamidomethylation as fixed modification and Met oxidation, N-terminal Gln/Glu cyclization, Asn/Gln deamidation, and Asn N-glycosylation as variable modifications. Mass tolerance values for peptide matches were set to 10 ppm and 0.05 Da for precursor and fragment ions, respectively. A homemade glycan database containing common biantennary structures and typical plant N-linked glycoforms was used for the identification of glycopeptides. Score thresholds for accepting peptide and glycopeptide identifications were Byonic^™^ score > 150. Glycopeptide identifications were manually validated to assign the glycoforms. The chromatographic area corresponding to glycopeptides and unmodified species was calculated by extracting the three most abundant ions observed in the corresponding spectra, both for the peptide EEQYNSTYR and the peptide TKPREEQYNSTYR containing two missed cleavages. The relative percentage of each glycoform was calculated as the ratio between the extracted ion area of the glycopeptide and the total extracted ion area of unmodified species and all corresponding glycopeptides. Two technical replicates were analyzed for each antibody sample.

### Virus neutralization assays

To evaluate the neutralization activity of mAbJ08 against the original SARS-CoV-2 isolated in Wuhan, China, a cytopathic effect (CPE)-based neutralization assay was performed (Andreano et al., 2021). Briefly, 100 median tissue culture infectious doses (100 TCID_50_) of SARS-CoV-2 authentic viruses were co-incubated with dilutions of mAb formulations for 1 h at 37°C in a 5% CO_2_ atmosphere (two-fold serial dilutions of mAbs starting at a concentration of 4 μg/mL). Next, the virus-mAbJ08 mixture was added to the 96-well plate containing sub-confluent Vero E6 cell monolayer, and the plates were incubated for 72 h at 37°C in a 5% CO_2_ atmosphere. After incubation, plates were examined for CPE by means of an inverted optical microscope. Non-infected Vero E6 cells and cells infected with the SARS-CoV-2 virus were used as negative and positive controls, respectively. Experiments were performed in technical duplicates, and positive and negative controls were used in each well as previously described (Andreano et al., 2021).

### Statistical analysis

Statistical analysis was performed using GraphPad Prism for Windows (GraphPad Software, La Jolla, CA, USA, www.graphpad.com). The normality of the data was tested using D'Agostino-Pearson omnibus normality test. A two-tailed Student's *t*-test Welch's correction for unpaired samples or Tukey-corrected one-way ANOVA was used, and an α-level of 0.05 was used as the threshold. *P*-values < 0.05 were considered to be statistically significant. *F*-test was used to compare variances.

## Results

### Transient expression of SARS-CoV-2 nAbs in *N. benthamiana*

**Figure 1:**
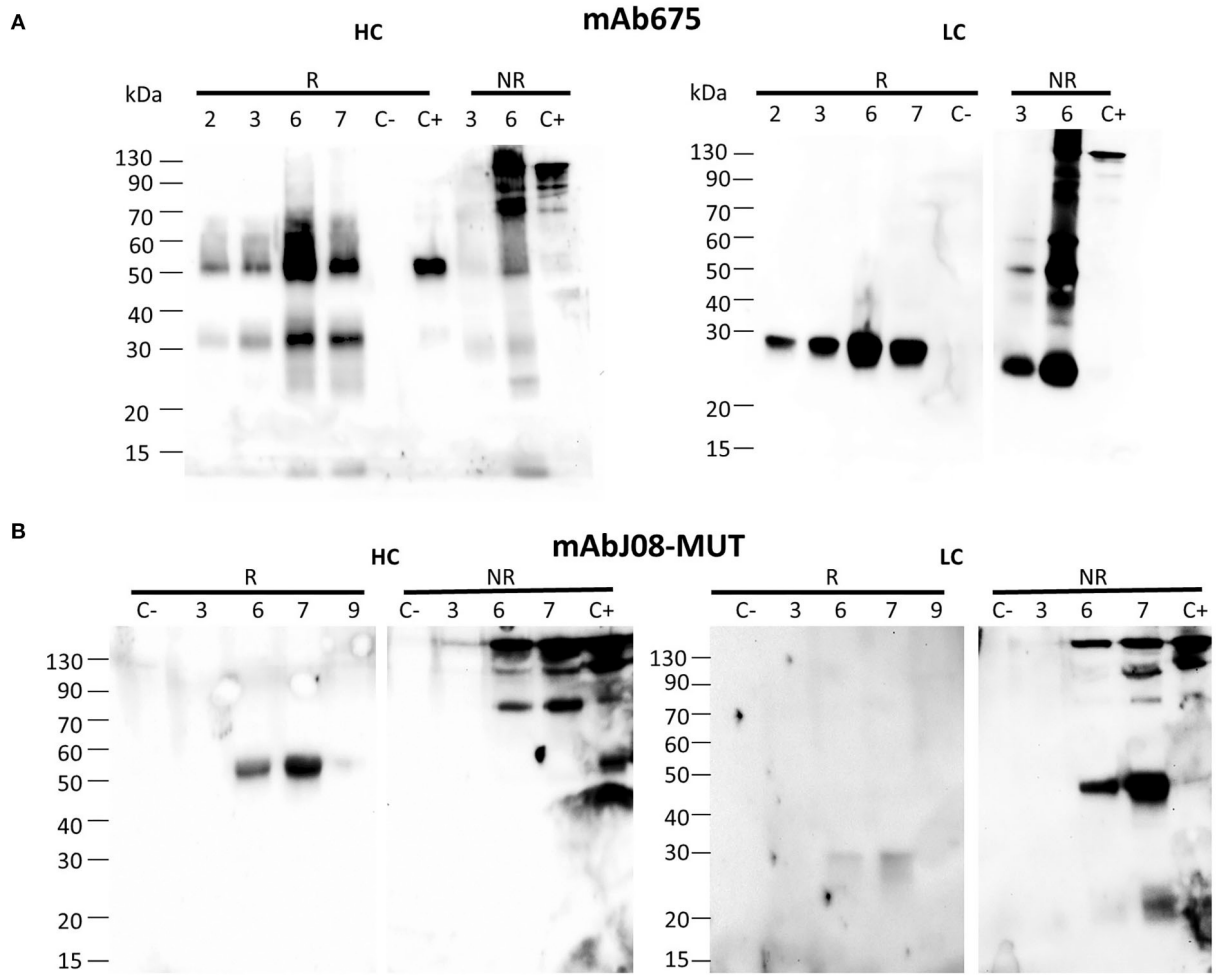
Accumulation of anti-SARS-CoV-2 nAbs in agroinfiltrated FX-KO *N. benthamiana* plants. Western blot analysis of extracts of *N. benthamiana* plants syringe co-agroinfiltrated with *A. tumefaciens* strains harboring the HC and LC of either mAb675 **(A)** or mAbJ08-MUT **(B)**. Samples obtained from leaves (taken at 2, 3, 6, or 7 d.p.i.) were separated on reducing (R) or non-reducing (NR) 12% SDS-PAGE. Plant extracts were normalized for total soluble protein (TSP) content; 10 μg of TSP was loaded for each sample. Extracts from plants infiltrated with only P19 silencing suppressor were used as negative control (C-). As positive control (C+), 50 ng of purified human IgG1 mAbRTX was used. Detection was performed with an anti-human γ chain (HC) or anti-human κ chain (LC) HRP-conjugated antibody.

The antibody expression was obtained in glycoengineered FX-KO *N. benthamiana* plants grown in soil infiltrated using a syringe. Analysis of the HC under reducing conditions (R) revealed the presence of a band at about 50 kDa, corresponding to the monomeric HC in both antibody samples (Figures 1A,B). In the case of mAb675, an additional band migrating at about 30 kDa was also visible, probably corresponding to a degradation fragment. Analysis under non-reducing conditions (NR) showed the presence of a band of approximately 150 kDa in both antibodies corresponding to the assembled immunoglobulin; a faint band of lower molecular mass (approx. 70 kDa) was also visible. Conversely, a band migrating at ~50 kDa was visible only in the mAb675 sample (Figure 1A).

Analysis of the LC under reducing conditions (R) revealed the presence of a single band at about 25 kDa, corresponding to the monomeric protein, which was very intense for mAb675, while very faint in the case of the mAbJ08-MUT sample. Under non-reducing conditions (NR), the presence of a strong band at the expected size of 150 kDa, corresponding to the assembled antibody, was observed in both samples. An additional band migrating at approximately 45 kDa was also detected, which was more evident in the case of the mAbJ08-MUT sample; this band probably corresponds to the formation of the LC dimer. Only in the case of mAb675, a strong band migrating at 25 kDa was also observed, which indicated the presence of unassembled monomeric LC. Both R and NR analyses of HC and LC revealed that the highest accumulation of mAb675 was reached at 6 d.p.i., while that of mAbJ08-MUT was obtained at 7 d.p.i (Figures 1A,B).

### Accumulation of nAbs in vacuum-agroinfiltrated plants grown in soil or in a hydroponic system

**Figure 2:**
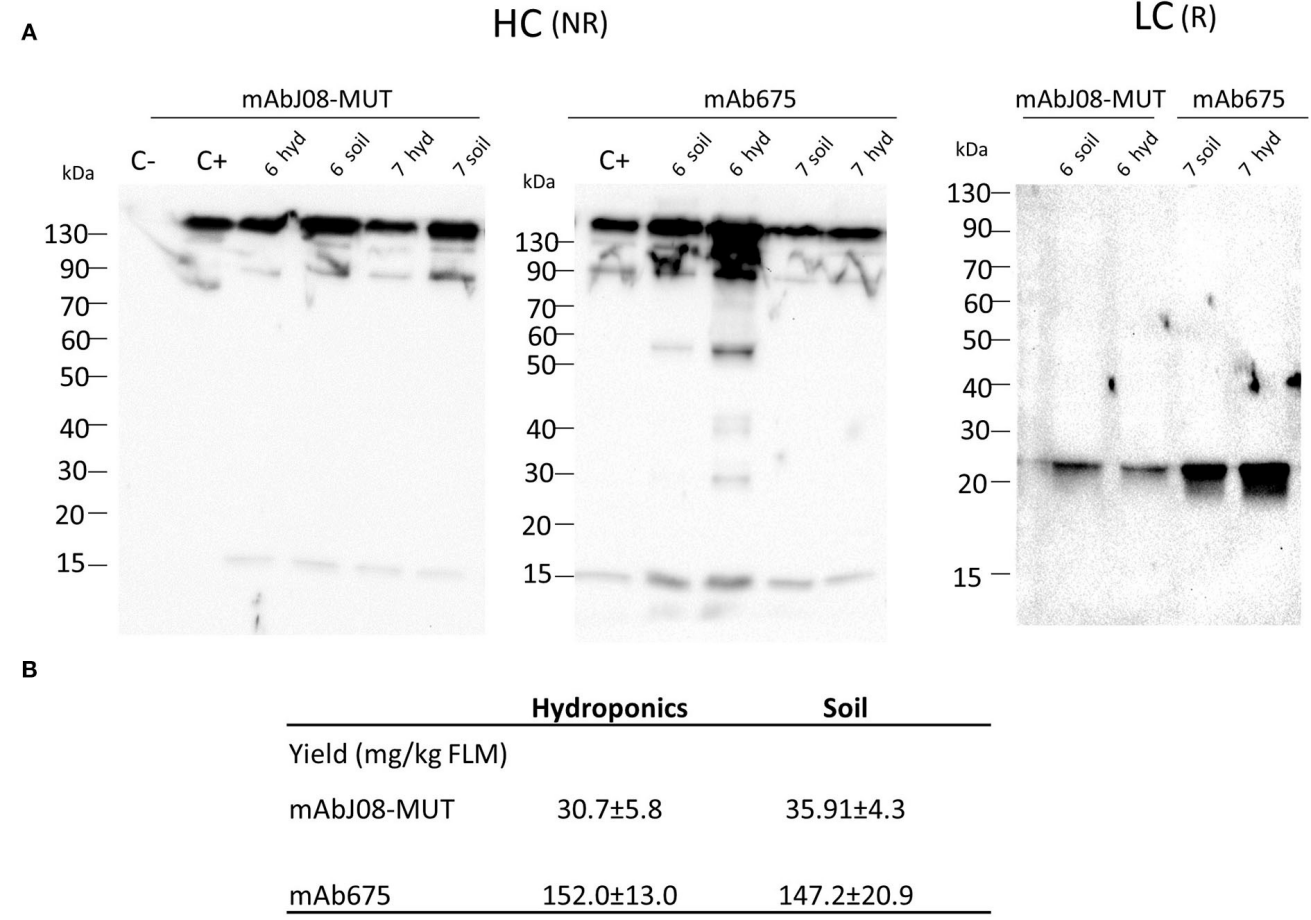
Accumulation of nAbs in vacuum-agroinfiltrated plants grown in soil or in a hydroponic system. **(A)** Western blot analysis of vacuum-agroinfiltrated FX-KO *N. benthamiana* plants producing mAbJ08-MUT and mAb675. Leaves from plants grown hydroponically (hyd) on rockwool substrate or in soil (soil) were collected on day 6 or 7 post-infiltration. Extracts normalized for total soluble protein (TSP) content were separated on reducing (R) or non-reducing (NR) 12% SDS-PAGE; 10 μg of TSP was loaded for each sample. C-: Extracts from plants infiltrated with only P19 silencing suppressor; C+: 50 ng of human IgG1 mAbRTX. Detection was performed with an anti-human γ chain (HC) or anti-human κ chain (LC) HRP-conjugated antibody. **(B)** Accumulation yield per kg of fresh leaf mass (FLM) of nAbs was calculated by quantitative double-antibody sandwich ELISA; reported values are the mean ± standard deviation (SD) (*n* = 3). Values of samples mAbJ08-MUT Hydroponics, mAbJ08-MUT Soil, mAb675 Hydroponics, and mAb675 Soil had coefficient of variations of 18.93, 12.02, 8.57, and 14.2%, respectively. *P* < 0.05 for mAbJ08-MUT Hydroponics compared to mAb675 Hydroponics and for mAbJ08-MUT Soil vs. mAb675 Soil. No significant difference was found for mAbJ08-MUT Hydroponics vs. mAbJ08-MUT Soil and mAb675 Hydroponics vs. mAb675 Soil (Unpaired two-tailed Student's *t*-test Welch's correction and Tukey-corrected one-way ANOVA).

Western blot analysis of leaf extracts of plants expressing mAbJ08-MUT using an anti-HC antibody under non-reducing conditions showed a major band migrating at 150 kDa corresponding to the assembled antibody (Figure 2A). Similar results were obtained for the extracts of the plants expressing mAb657, where a 50 kDa band corresponding to a degradation product was also visible. Analysis of LC expression under reducing conditions evidenced for both antibodies a band at 25 kDa corresponding to the monomeric LC, but with a reduction in the corresponding intensity in the mAbJ08-MUT samples, thus indicating lower expression levels in this case. Accumulation of nAbs was calculated by quantitative DAS-ELISA, with approximate yields of ~150 mg/kg of fresh leaf mass (FLM) for mAb675 and ~35 mg/kg for mAbJ08-MUT (Figure 2B). Overall, no difference in the band intensity and accumulation yields of the two antibodies was observed between plants grown hydroponically (hyd) and those grown in soil (soil).

### Effect of LC expression levels on the accumulation of nAbs

**Figure 3:**
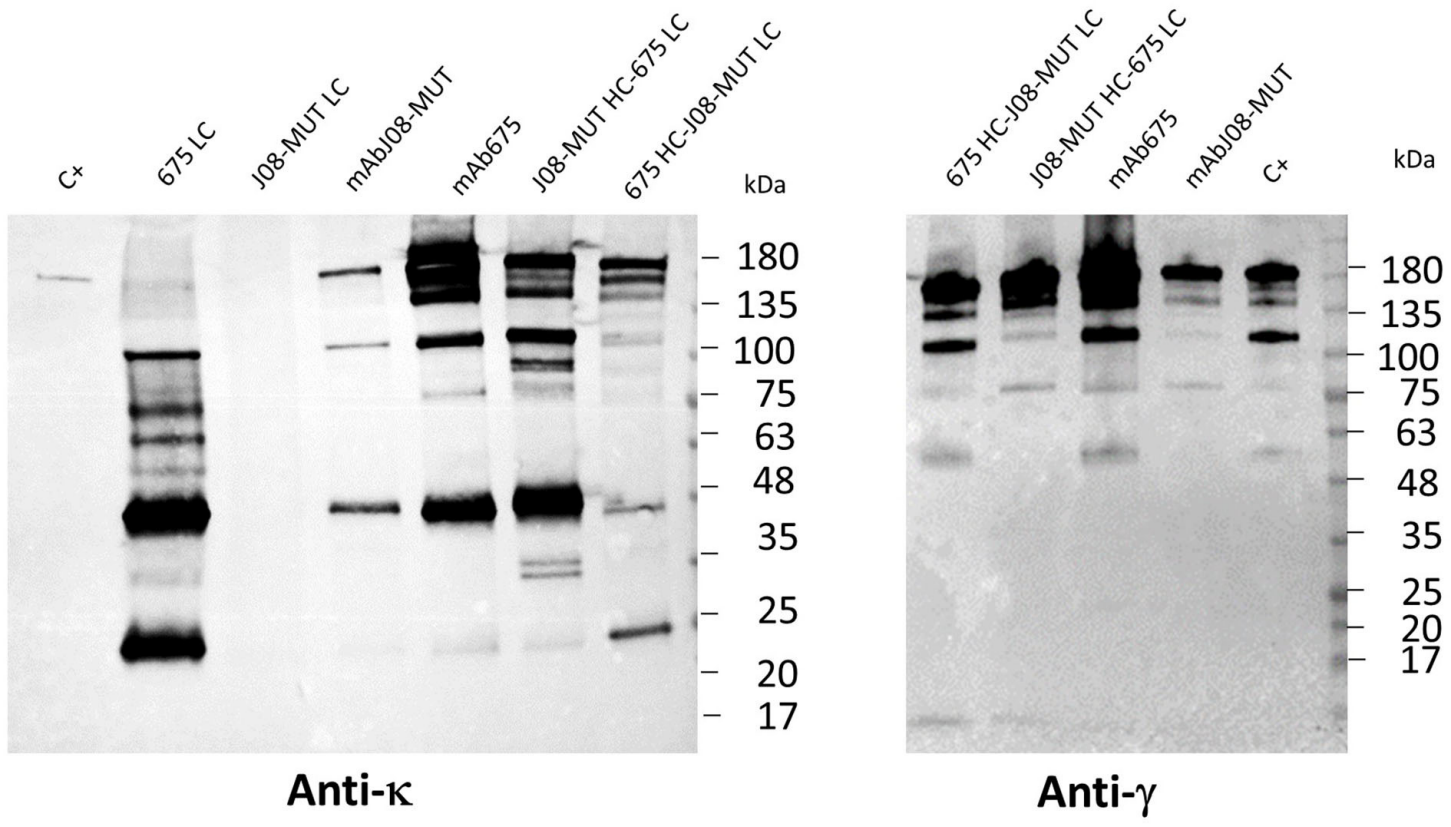
Effect of LC expression levels on the accumulation of antibodies. Western blot analysis of leaves agroinfiltrated with single LCs of mAbJ08-MUT and mAb675 or with HC and LC combinations of the two nAbs (J08-MUT-HC-675 LC and 675 HC-J08-MUT LC). Agroinfiltrated leaves were collected on day 6 post-infiltration. Extracts normalized for total soluble protein (TSP) content were separated on non-reducing (NR) 12% SDS-PAGE; 15 μg of TSP was loaded for assembled antibody samples, while 45 μg of TSP was loaded for the two 675 LC and J08-MUT-LC samples. C+: 50 ng of purified human IgG1 mAbRTX. Detection was performed with an anti-human γ chain (HC) or anti-human κ chain (LC) HRP-conjugated antibody.

In order to study the differences in the expression behavior of mAb675 and mAbJ08-MUT, and the possible impact of LC expression efficacy on the yield of the complete antibody, each antibody LC and two chimeric Abs obtained by swapping HC and LC between mAb675 and mAbJ08-MUT were produced in plants by syringe agroinfiltration. Leaf extracts were separated on NR 12% SDS-PAGE and analyzed by Western blot with an anti-human κ chain (LC) or an anti-human γ chain (HC) HRP-conjugated antibody (Figure 3). Analysis of the κ chain revealed that accumulation of LC alone could only be detected for mAb675 (675 LC) with two major bands, one at 25 kDa corresponding to unassembled monomeric LC and a stronger one at about 50 kDa associated with the formation of the dimer. Analysis of mAbJ08-MUT and mAb675 with interchanged HC and LC combinations (675 HC-J08-MUT LC and J08-MUT-HC-675 LC) showed the presence of a band in all cases at about 150 kDa corresponding to the assembled antibodies. However, a higher accumulation of the assembled antibodies was observed when the HC of mAb675 and J08-MUT-HC was expressed in combination with the LC of mAb675. This finding was evident when the parallel analysis of the HC of mAb675 and J08-MUT expressed with the LC of J08-MUT was performed, which also resulted in the accumulation of unassembled LC, as evidenced by the presence of a strong band at about 50 kDa. The same results were obtained when the samples were revealed with an anti-γ chain antibody, indicating the positive effect of the higher expression efficiency of mAb675 LC on the overall yield of both chimeric Abs.

### Purification and characterization of the plant-derived antibodies

Plant extracts were typically prepared from 40-g batches of leaves agroinfiltrated with either mAbJ08-MUT or mAb657. Purification was performed using a HiTrap^™^ FF Protein-A column. The average yield was 16 mg/kg FLM or 70 mg/kg FLM for mAbJ08-MUT and mAb657, respectively.

**Figure 4:**
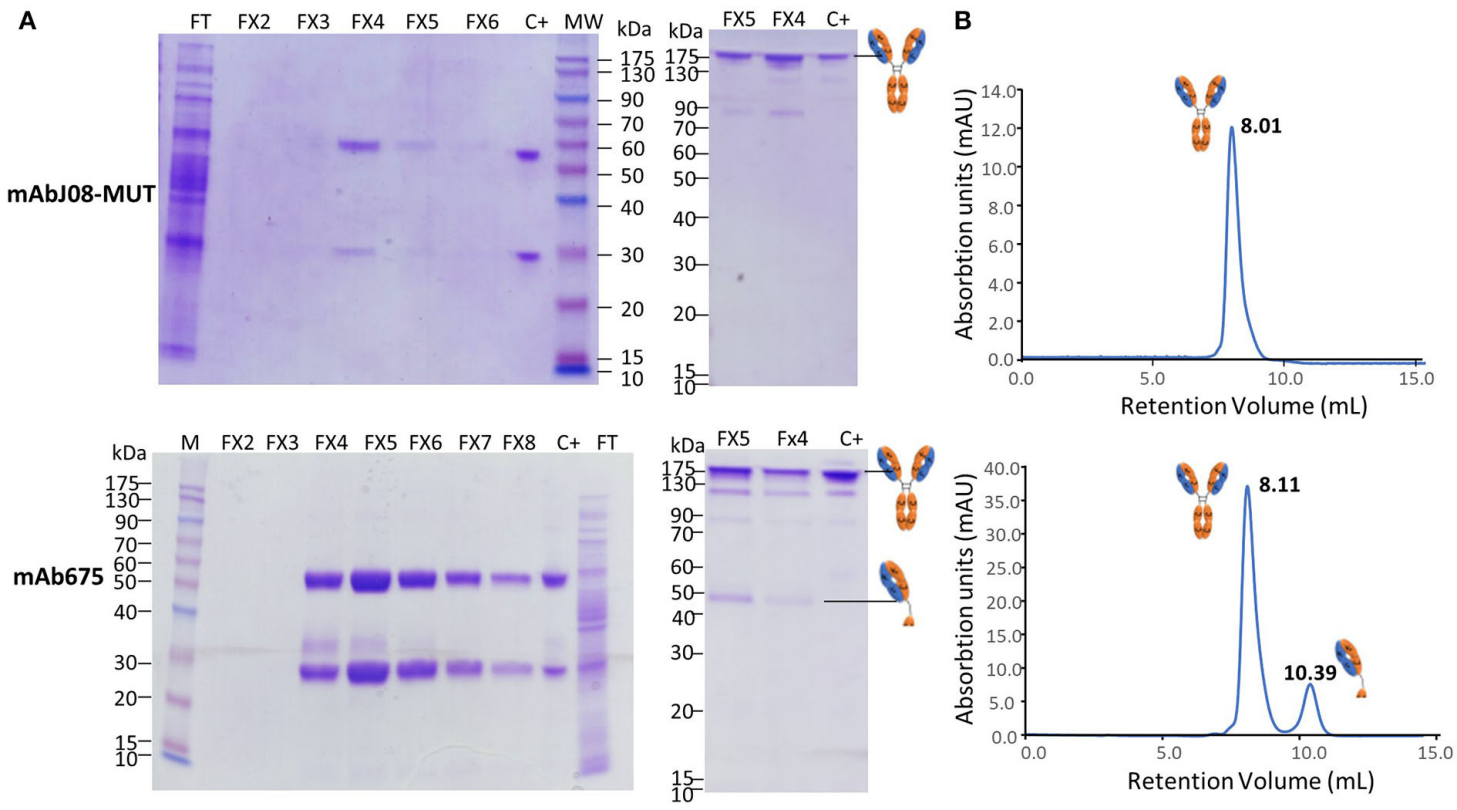
Purification and characterization of the plant-derived antibodies. AntiSARS-CoV-2 nAbs were purified by protein-A affinity chromatography and eluted fractions (FX-10 μL per lane) were separated by 12% SDS-PAGE under reducing (left side) and non-reducing conditions (right side), followed by Coomassie blue staining **(A)**. FT: flow-through of protein-A column; C+: purified human IgG1 mAbRTX used as a reference (2 μg). **(B)** Size-exclusion chromatography was performed on a Superdex^™^ 75 10/300 GL column, as described in the Section Materials and methods. The retention volumes (mL) of the peaks obtained and the schematic representation of the molecules contained in the eluted peaks are indicated.

A typical 12% SDS-PAGE analysis of the purified antibodies under reducing and non-reducing conditions is shown in Figure 4A. SDS-PAGE of eluted fractions under reducing conditions showed the expected bands for both antibodies migrating at 50 and 25 kDa corresponding to HC and LC, respectively. An additional faint band migrating at about 30 kDa was visible in the mAb657 fractions, probably corresponding to an HC degradation product. Non-reducing SDS-PAGE evidenced the correct assembly of the antibodies (band at about 150 kDa); in the case of mAb675, a faint band migrating at 50 kDa was also detected, similar to what was observed in the Western blot analysis of crude extracts.

Assembly and purity of the antibodies were verified by size-exclusion chromatography using a Superdex S-75 column. This analysis revealed that mAbJ08-MUT was intact (major peak area ≥ 99% corresponding to the assembled antibody), eluting approximately at 8.01 mL. Conversely, mAb675 showed a major peak (about 81% of total area) corresponding to the intact antibody and a minor peak eluting at 10.39 mL (almost 18% of total area) that was associated with a product having a lower molecular mass (50 kDa), also observed in NR SDS-PAGE analysis (Figure 4B). In order to assign the nature of the mAb675 components migrating at 150 kDa and 50 kDa, the corresponding gel bands were excised and digested with trypsin; the resulting protein digests were then subjected to peptide mapping experiments by MALDI-TOF-MS analysis. The peptide coverage of the HC and LC amino acid sequences for these mAb675 products is reported in Supplementary Figure S2. Analysis of the identified peptides ascertained that the component migrating at 150 kDa corresponded to the intact antibody, while that eluting at ~50 kDa band lacked a significant polypeptide portion at the C terminus of HC (Supplementary Figure S2); the latter approximatively started at the beginning of the CH2 domain (^249^DTLMISRTPD). Similar peptide mapping experiments of mAbJ08-MUT confirmed the integrity of the recombinant product (data not shown).

In order to characterize the modification status of the unique putative N-glycosylation site occurring in intact mAb675 and mAbJ08-MUT (Supplementary Figure S2), the corresponding tryptic digests were also subjected to nanoLC-ESI-Q-Orbitrap-MS/MS analysis, followed by a database search of the resulting data. Assignment to specific N-linked glycan structures was based on mass fragmentation data. This experiment demonstrated the presence of a single glycosylation site in both mAb675 and mAbJ08-MUT, as deduced by the occurrence of the homologous glycopeptides TKPREEQYNSTYR and EEQYNSTYR observed in both digests. The estimation of the relative abundance of the different glycoforms was obtained by measuring the area of the extracted ions (XICs) of the identified glycopeptide species from nanoLC-ESI-MS chromatograms (Supplementary Figure S3). This analysis revealed the preponderant presence of an *N*-linked glycopeptide species bearing the complex glycan GlcNAc_4_Man_3_ and the absence of typical plant-type glycostructures, including α-1,3-fucose and β-1,2-xylose residues. A reduced percentage of non-glycosylated forms was also detected in both mAb675 and mAbJ08-MUT (Supplementary Figure S3).

### RBD recognition, RBD-binding competition with ACE2, and neutralization activity against SARS-CoV-2 of the plant-derived antibodies

**Figure 5:**
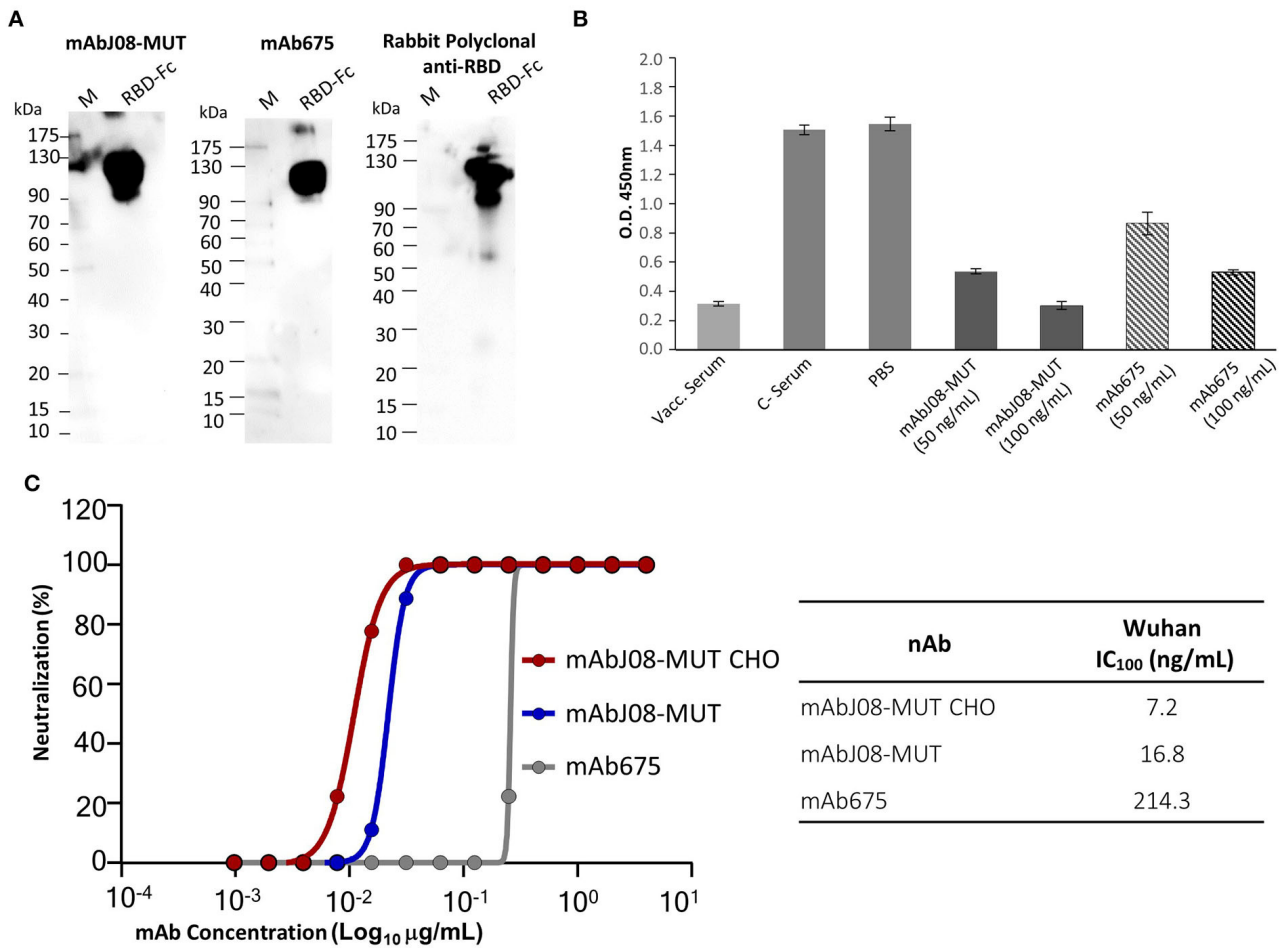
RBD recognition, RBD-binding competition with ACE2, and neutralization activity against SARS-CoV-2 of the plant-derived antibodies. **(A)** The plant-purified mAb675 and mAbJ08-MUT were assayed for binding to recombinant RBD in the Western blotting experiments. A commercial RBD fusion protein (25 ng) produced in HEK293 cells was separated on non-reducing 12% SDS-PAGE and assayed with both antibodies at a dilution of 200 ng/mL; C+: Commercial rabbit anti-RBD polyclonal antibodies were used as a control. **(B)** A competitive ELISA was used to assay the capacity of plant-produced nAbs to compete for the binding of ACE2 to RBD. The plate was coated with a SARS-CoV-2 RBD antigen. Samples with neutralizing antibodies were incubated with excess amounts of biotinylated ACE2, and any ACE2 bound to the RBD was assayed with Streptavidin-HRP conjugate antibody. Both nAbs were assayed at a concentration of 100 and 50 ng/mL; a serum sample from a vaccinated individual at 1:50 dilution was also assayed, together with pre-vaccine serum (C-) and PBS. Values are the mean ± SD (*n* = 3); Unpaired two-tailed Student's *t*-test, *p* = 0.0001 for mAbJ08-MUT 50 ng, mAbJ08-MUT 100 ng, and mAb675 100 ng vs. both C– Serum and PBS. *P* **=** 0.0007 and *P* **=** 0.0002 for mAb675 50 ng vs. PBS and C– Serum, respectively. **(C)** The neutralization curve for the plant-produced antibodies as well as the mAbJ08-MUT CHO produced in a mammalian cell line was calculated, and the percentage of viral neutralization against the authentic SARS-CoV-2 wild type as obtained for different mAb concentrations is reported. The calculated 100% inhibitory concentration (IC100) for the three nAbs is also shown for comparison.

Plant-purified mAb675 and mAbJ08-MUT were then assayed for binding to RBD by both Western blotting and ELISA techniques. Both antibodies (at a dilution of 200 ng/mL) recognized a recombinant RBD-mouse Fc fusion protein (dimeric form) in NR Western blot experiments, as demonstrated by the presence of a band migrating at the expected molecular mass (~110 kDa) (Figure 5A). Both nAbs at concentrations of 50 and 100 ng/mL, as well as serum samples from a vaccinated individual, showed a significant signal reduction compared to the corresponding controls, indicating a binding competition to RBD with ACE2 (Figure 5B). The two plant-produced antibodies were also tested to confirm the neutralization potency against the original Wuhan SARS-CoV-2 virus, by comparing their activity with the mAbJ08-MUT produced in CHO (mAbJ08-MUT CHO) (Figure 5C). The plant-produced mAbJ08-MUT maintained extremely high neutralization potency against SARS-CoV-2, showing an IC_100_ value of 16.8 ng/mL that was comparable to that of mAbJ08-MUT CHO (7.2 ng/mL). As expected, mAb675 showed a neutralization potency in the medium range (214.3 ng/mL).

## Discussion

Despite the positive results and the consequent approval of several SARS-CoV-2 neutralizing mAbs, the study and development of novel antibodies still remain a priority in COVID pandemics, considering the emergence of new variants of the virus presenting mutations in the spike protein. Therefore, the rapid development of new antibodies and their use in different combinations could prevent the possible failure of treatments due to the resistance of new variants to established neutralizing antibodies (Li et al., 2022). In this context, the use of plants could represent an ideal manufacturing approach allowing to rapidly produce different promising nAbs in sufficient quantities to be evaluated for therapeutic use. In fact, it has been calculated that it is possible to obtain a sufficient amount of product to enter the clinical testing in about 3 months compared to the 6 months required by using mammalian cells (Tusé et al., 2020).

In this study, we reported the production in plants and the characterization of two anti-SARS-CoV-2 mAbs, among which one derived from the human parent molecule isolated from a COVID-19 convalescent patient (mAbJ08) endowed with a picomolar affinity toward the spike protein RBD and an extremely potent virus-neutralizing activity (Andreano et al., 2021). We transiently expressed both antibodies in plants and observed an accumulation peak 6–7 days after agroinfiltration, as already described in the literature in the case of anti-tumor antibodies expressed using the same transient expression strategy (Lombardi et al., 2010). Expression analysis revealed a strong difference in LC expression between the two antibodies, with mAbJ08-MUT exhibiting lower accumulation levels compared to mAb675, and the formation of LC dimers. This behavior was reflected in a strong difference in the expression levels of assembled antibodies, with mAb675 showing a three-fold higher accumulation compared to mAbJ08-MUT. The positive effect of LC expression levels on mAb accumulation was also demonstrated by the higher yields observed for the chimeric antibody obtained by the assembly of mAb675 LC with mAbJ08-MUT-HC. All these observations were in agreement with the previous evidence that LC levels in mammalian cell culture medium reflect recombinant monoclonal antibody productivity and quality (Bhoskar et al., 2013; Pybus et al., 2014; Ishii et al., 2016). Indeed, it was previously reported that in mammalian cells also mAbJ08-MUT-LC levels were low (Andreano et al., 2021).

Nevertheless, reported yields of about 35 mg/kg for plant-produced mAbJ08-MUT were somehow comparable or higher than those previously described for transiently expressed anti-SARS-CoV-2 mAbs B38 and H4 (4 and 35 mg/kg, respectively) using a viral vector (Shanmugaraj et al., 2020). Western blot analysis highlighted in mAb675 the presence of bands corresponding to degradation products migrating at about 50 kDa in non-reducing conditions, and at 25 kDa in reducing conditions. Antibody purification by protein-A affinity chromatography showed average yields of 16 mg/kg FLM or 70 mg/kg FLM for mAbJ08-MUT and mAb657, respectively, corresponding to about 50% loss during the recovery process. It is possible that major antibody loss occurs during extraction and clarification prior to affinity column purification as observed in previous work using a similar extraction and purification protocol for a human IgG1 produced by transient expression in *N. benthamiana* plants (Lombardi et al., 2010). The *in planta* degradation of antibodies is a known phenomenon that has been extensively studied and is due to the presence of plant-specific proteases capable of recognizing and fragmenting the HC of human IgG1 (Donini et al., 2015; Hehle et al., 2015). Mass spectrometry analysis allowed us to determine the nature of these bands, suggesting that the proteolytic cleavage took place within the CH2 domain, close to the hinge region, leading to the formation of Fab fragments. The susceptibility of antibodies to proteolytic cleavage at the level of the hinge region and the CH2 domain is well-known. In particular, it was shown to be caused by specific classes of proteases, such as cysteine and serine proteases (Jutras et al., 2019), and although several approaches have been studied to avoid this degradation in plants, the effects on some antibodies are very important (Donini et al., 2015; Jutras et al., 2016). In fact, degradation can compromise the yield and final quality of the purified product, as demonstrated for the antitumor mAb H10 for which 90% of the purified plant molecule was degraded (Villani et al., 2009). However, in the case of mAb675, size-exclusion chromatography showed that only a low percentage of the antibody (~17%) is degraded, while mAbJ08-MUT resulted to be less prone to proteolytic degradation, with more than 99% of the antibody being intact. The presence of five-point mutations in the Fc region of mAbJ08, which were introduced to increase the half-life and abrogate binding to Fc receptors, could partially explain the observed resistance to proteolytic degradation.

Different cultivation strategies can have a major influence on the performances of plant “biofactories” used for the production of biopharmaceuticals (Huebbers and Buyel, 2021). The conditions like the type of irrigation and plant growth support may have an influence on plant physiology and possibly on the accumulation and quality of the biopharmaceutical product. In this work, we demonstrated that the type of cultivation (hydroponics on rockwool compared to soil) has no effect on the yield and quality of both nAbs. An important aspect of plant-produced antibodies is their glycosylation profile, which can affect their biological stability and function. In order to obtain antibodies with a homogeneous human-compatible glycosylation profile, in this study, we used genome-edited plants in which the genes encoding for the enzymes responsible for adding typical plant sugars (α1,3-fucose and β1,2-xylose) were inactivated (Jansing et al., 2019). In fact, it has been hypothesized, but never fully demonstrated, that the use of biopharmaceuticals produced in plants containing typical plant sugars may trigger an adverse immune response in humans (Jin et al., 2008). Glycosylation analysis allowed us to verify that both nAbs produced in hydroponically grown *N. benthamiana* share a homogeneous glycan profile with N-linked complex-type oligosaccharides (GlcNAc4Man3) and no detectable typical plant sugars, thus demonstrating that it is possible to obtain molecules with a 'humanized' glycosylation profile in suitably engineered plants. Comparing the glycan profile of the nAbs with that of the HIV-neutralizing mAb 2G12 previously produced in the same glyco-engineered plant line traditionally grown on soil (Jansing et al., 2019), no significant difference can be evidenced, and all antibodies showed the absence of xylose and fucose residues and the major glycan species GlcNAc4Man3 representing about 60 % of total sugars.

Both plant-purified nAbs showed their ability to bind the SARS-CoV-2 RBD both in their denatured form in the Western Blot analysis and in their native form in a competitive ELISA. The latter assay also allowed to determine whether the plant-derived antibodies interfere with the binding of RBD to ACE2, with a 65 and 44% reduction of ACE2 binding for mAbJ08-MUT and mAb675 (both at 50 ng/mL concentration), respectively. Furthermore, the virus neutralization assay confirmed the high potency of the plant-produced mAbJ08-MUT with IC_100_ values in the range of 10 ng/mL, which were very close to those of the antibody produced in mammalian cells, while mAb675 showed much lower values (200 ng/mL), in line with previously reported data for the same antibody produced in HEK cells (Wang et al., 2021).

Only a few SARS-CoV-2 nAbs have been produced in plants so far. Two human monoclonal antibodies against SARS-CoV-2 (B38 and H4) were produced by transient expression using viral vectors in *N. benthamiana* leaves (Shanmugaraj et al., 2020) and showed neutralization efficacy *in vitro* against the original SARS-CoV-2 isolated in Wuhan at neutralization titer (NT) values of 5,450 ng/mL and 492 ng/mL, respectively. Later on, four variants of the neutralizing H4 antibody, in which the same variable domain was fused to the constant domains of the four subclasses of IgG present in human serum, were produced in plants (Kallolimath et al., 2021). These recombinant products were compared for their efficacy in the response to SARS-CoV-2 infection; interestingly, it was observed that H4-IgG3 exhibited up to 50-fold superior neutralization potency (IC_100_ values of 1,000 ng/mL), due to the unique structural features of this antibody subclass, which shows increased avidity for virus-displayed spike proteins. A table reporting neutralization potencies of nAbs obtained in plants and mammalian cells is presented (Table 1). Differences in the IC values of the same antibody obtained either in plants or mammalian cells are evident for nAbs CA1 and CB6 (Jugler et al., 2022). In some cases, it is difficult to directly compare data between the publications due to differences in the IC calculations, but it is evident that mAbJ08-MUT produced both in plants and mammalian cells is the only nAb endowed with potencies in the 10 ng/mL range.

**Table 1:** Comparison of neutralization potencies of the same nAbs obtained in plants and mammalian cells.

**nAb**	**Neutralization potency**	
	**Plant-produced nAb**	**Mammalian cell-produced nAb**
675	IC_100:_ 214.3 ng/mL (this study)	IC_50_: 19.5 ng/mL (Wang et al., 2021) IC_90_: 111.6 ng/mL (Wang et al., 2021)
J08-MUT	IC_100:_ 16.8 ng/mL (this study)	IC_100:_ 7.2 ng/mL (this study)
CA1	IC_50_: 1390 ng/mL (Jugler et al., 2022)	IC_50_: 4981 ng/mL (Shi et al., 2020)
CB6	IC_50_: 139 ng/mL (Jugler et al., 2022)	IC_50_: 835 ng/mL (Shi et al., 2020)
B38	NT40 at 5450 ng/mL (Shanmugaraj et al., 2020)	IC_50:_ 177 ng /mL (Wu et al., 2020)
H4	NT 640 at 492 ng/mL (Shanmugaraj et al., 2020)	IC_50:_ 896 ng/mL (Wu et al., 2020)
H4-IgG3	IC_100:_ 1000 ng/mL (Kallolimath et al., 2021)	IC_50:_ 896 ng/mL (Wu et al., 2020)

In these studies, potencies were calculated using cytopathic effect (CPE)-based neutralization assays on Vero E6 cells and were expressed as inhibitory concentration (IC) except for the work by Shanmugaraj et al. in which neutralization titer (NT) was reported. The IC is defined as the antibody concentration at which the viral replication has been reduced by 50% (IC_50_), 90% (IC_90_), or 100% (IC_100_).

Extremely potent nAbs display several advantages, such as the possibility to reduce the dosage necessary to reach prophylactic and therapeutic efficacy (as low as 50–100 mg per single administered dose) and the possibility of using alternative administration strategies, such as the intramuscular route, as already demonstrated for mAbJ08-MUT in the clinical Phase I study (Lanini et al., 2022). All these aspects may reduce treatment costs, allowing this therapy to be more affordable, especially for low-income countries. In this context, the plant production systems whose main limit is represented by the overall lower yields, compared to traditional manufacturing systems, may gain a competitive advantage (Schillberg and Finnern, 2021).

In conclusion, we have described here the successful expression of an Fc-engineered SARS-CoV-2 nAb with a human-like glycosylation profile endowed with very potent neutralizing activity in plants, comparable to that of its cognate mammalian cell-derived counterpart. We also demonstrated that nAbs can be efficiently produced in plants hydroponically grown on the inorganic substrate and that the LC yield can have a major impact on overall antibody accumulation. Thus, plant expression platforms may represent a convenient rapid production system of such potent SARS-CoV-2 nAbs to be used both in therapy and diagnostics.

## Data availability statement

The original contributions presented in the study are included in the article/Supplementary material, and further inquiries can be directed to the corresponding author/s.

## Author contributions

RF performed the experiments and data analysis and wrote sections of the manuscript. CM contributed to the conception of the study and helped perform experiments. MV, CL, CC, EA, IP, and AMS carried out experiments, performed data analyses, and wrote sections of the manuscript. SB, RR, and AS helped in revising the manuscript. MD conceived, designed the study, performed the experiments, and wrote the manuscript. All the authors contributed to the manuscript revision, read, and approved the submitted version.

## Funding

This work has been carried out with ENEA internal funding.

## Conflict of interest

RR is an employee of the GSK group of companies. EA, IP, and RR are listed as inventors of full-length human monoclonal antibodies described in Italian patent applications n. 102020000015754 filed on 30 June 2020, 102020000018955 filed on 3 August 2020, and 102020000029969 filed on 4 December 2020, and the international patent system number PCT/IB2021/055755 filed on 28 June 2021. The remaining authors declare that the research was conducted in the absence of any commercial or financial relationships that could be construed as a potential conflict of interest.

## Publisher's note

All claims expressed in this article are solely those of the authors and do not necessarily represent those of their affiliated organizations, or those of the publisher, the editors and the reviewers. Any product that may be evaluated in this article, or claim that may be made by its manufacturer, is not guaranteed or endorsed by the publisher.
