# Supplementary material for: Production of two SARS-CoV-2 neutralizing antibodies with different potencies in Nicotiana benthamiana
Source: Front Plant Sci. 2022 Sep 5;13:956741. doi: 10.3389/fpls.2022.956741 (PMC9484322; doi:10.3389/fpls.2022.956741)
Supplement: Supplementary file 1 [file Presentation_1.PPTX]

## Slide 1
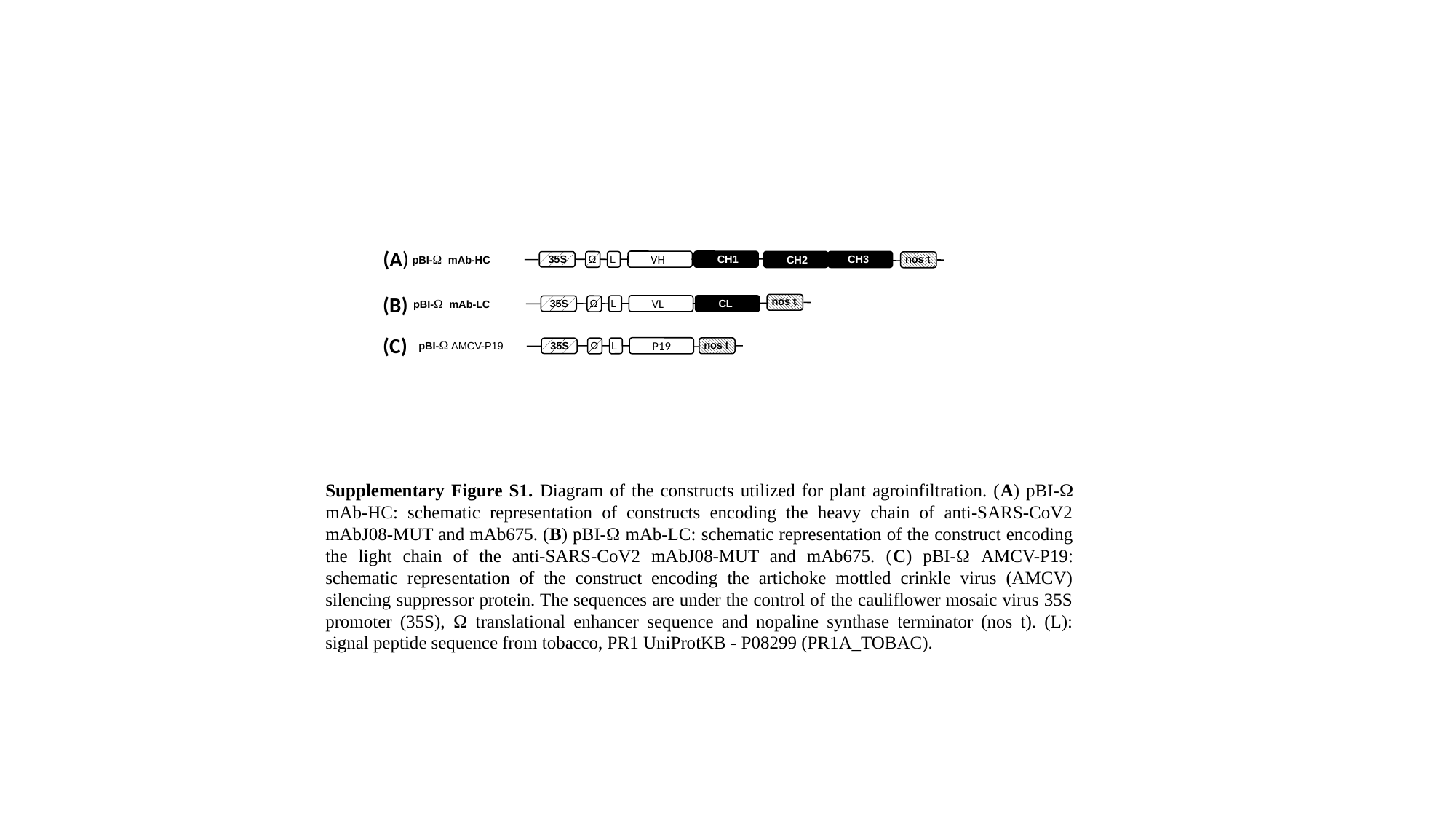

(A)
pBI-Ω mAb-HC
VH
nos t
CH3
35S
Ω
L
CH2
 CH1
(B)
nos t
pBI-Ω mAb-LC
VL
CH3
35S
Ω
L
 CL
(C)
pBI-Ω AMCV-P19
nos t
P19
35S
Ω
L
Supplementary Figure S1. Diagram of the constructs utilized for plant agroinfiltration. (A) pBI-Ω mAb-HC: schematic representation of constructs encoding the heavy chain of anti-SARS-CoV2 mAbJ08-MUT and mAb675. (B) pBI-Ω mAb-LC: schematic representation of the construct encoding the light chain of the anti-SARS-CoV2 mAbJ08-MUT and mAb675. (C) pBI-Ω AMCV-P19: schematic representation of the construct encoding the artichoke mottled crinkle virus (AMCV) silencing suppressor protein. The sequences are under the control of the cauliflower mosaic virus 35S promoter (35S), Ω translational enhancer sequence and nopaline synthase terminator (nos t). (L): signal peptide sequence from tobacco, PR1 UniProtKB - P08299 (PR1A_TOBAC).
