# Supplementary material for: Production of two SARS-CoV-2 neutralizing antibodies with different potencies in Nicotiana benthamiana
Source: Front Plant Sci. 2022 Sep 5;13:956741. doi: 10.3389/fpls.2022.956741 (PMC9484322; doi:10.3389/fpls.2022.956741)
Supplement: Supplementary file 2 [file Presentation_2.PPTX]

## Slide 1
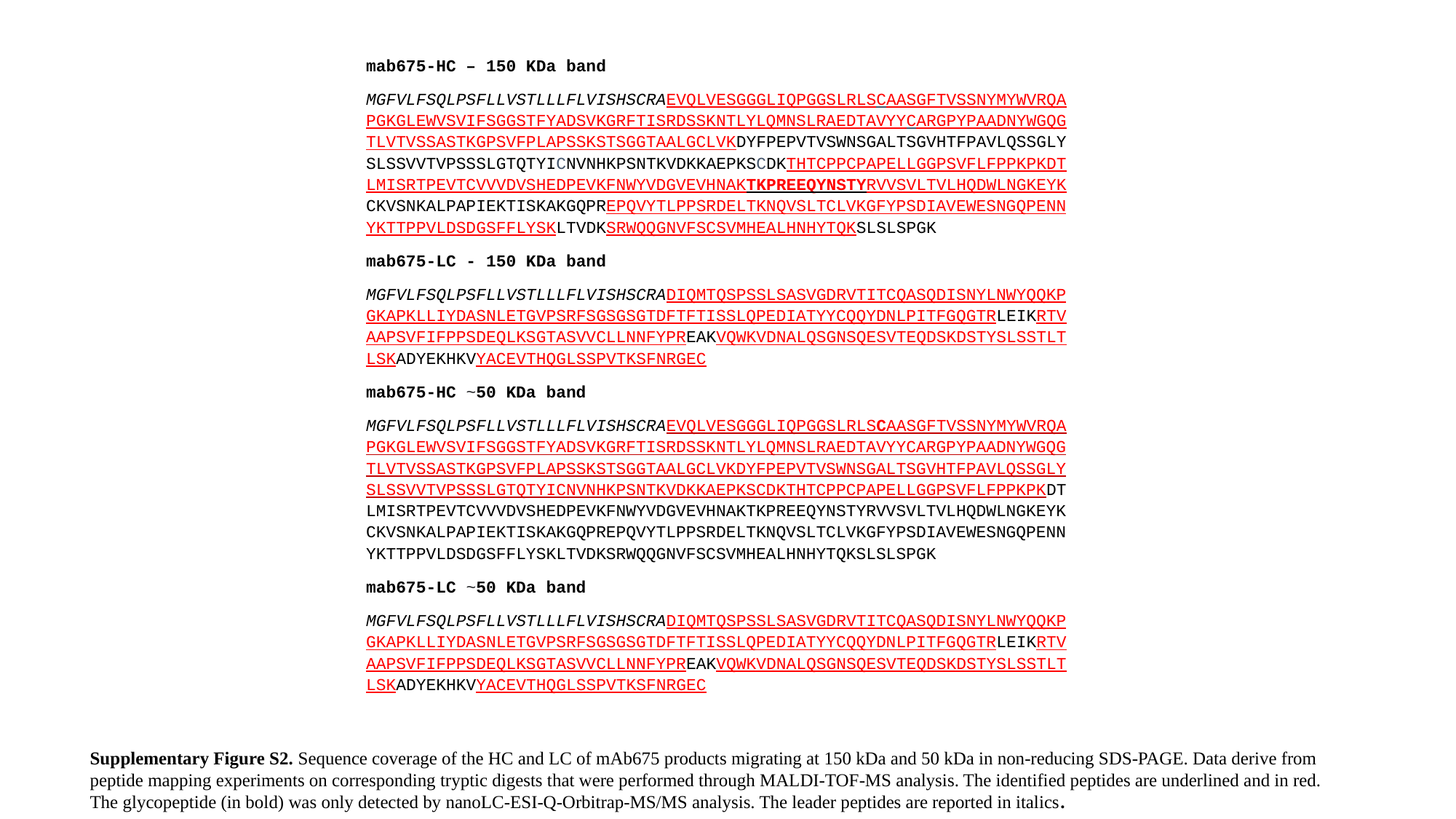

mab675-HC – 150 KDa band
MGFVLFSQLPSFLLVSTLLLFLVISHSCRAEVQLVESGGGLIQPGGSLRLSCAASGFTVSSNYMYWVRQAPGKGLEWVSVIFSGGSTFYADSVKGRFTISRDSSKNTLYLQMNSLRAEDTAVYYCARGPYPAADNYWGQGTLVTVSSASTKGPSVFPLAPSSKSTSGGTAALGCLVKDYFPEPVTVSWNSGALTSGVHTFPAVLQSSGLYSLSSVVTVPSSSLGTQTYICNVNHKPSNTKVDKKAEPKSCDKTHTCPPCPAPELLGGPSVFLFPPKPKDTLMISRTPEVTCVVVDVSHEDPEVKFNWYVDGVEVHNAKTKPREEQYNSTYRVVSVLTVLHQDWLNGKEYKCKVSNKALPAPIEKTISKAKGQPREPQVYTLPPSRDELTKNQVSLTCLVKGFYPSDIAVEWESNGQPENNYKTTPPVLDSDGSFFLYSKLTVDKSRWQQGNVFSCSVMHEALHNHYTQKSLSLSPGK
mab675-LC - 150 KDa band
MGFVLFSQLPSFLLVSTLLLFLVISHSCRADIQMTQSPSSLSASVGDRVTITCQASQDISNYLNWYQQKPGKAPKLLIYDASNLETGVPSRFSGSGSGTDFTFTISSLQPEDIATYYCQQYDNLPITFGQGTRLEIKrtvaapsvfifppsdeqlksgtasvvcllnnfypreakvqwkvdnalqsgnsqesvteqdskdstyslsstltlskadyekhkvyacevtHqgLSSPvtksfnrgec
mab675-HC ~50 KDa band
MGFVLFSQLPSFLLVSTLLLFLVISHSCRAEVQLVESGGGLIQPGGSLRLSCAASGFTVSSNYMYWVRQAPGKGLEWVSVIFSGGSTFYADSVKGRFTISRDSSKNTLYLQMNSLRAEDTAVYYCARGPYPAADNYWGQGTLVTVSSASTKGPSVFPLAPSSKSTSGGTAALGCLVKDYFPEPVTVSWNSGALTSGVHTFPAVLQSSGLYSLSSVVTVPSSSLGTQTYICNVNHKPSNTKVDKKAEPKSCDKTHTCPPCPAPELLGGPSVFLFPPKPKDTLMISRTPEVTCVVVDVSHEDPEVKFNWYVDGVEVHNAKTKPREEQYNSTYRVVSVLTVLHQDWLNGKEYKCKVSNKALPAPIEKTISKAKGQPREPQVYTLPPSRDELTKNQVSLTCLVKGFYPSDIAVEWESNGQPENNYKTTPPVLDSDGSFFLYSKLTVDKSRWQQGNVFSCSVMHEALHNHYTQKSLSLSPGK
mab675-LC ~50 KDa band
MGFVLFSQLPSFLLVSTLLLFLVISHSCRADIQMTQSPSSLSASVGDRVTITCQASQDISNYLNWYQQKPGKAPKLLIYDASNLETGVPSRFSGSGSGTDFTFTISSLQPEDIATYYCQQYDNLPITFGQGTRLEIKrtvaapsvfifppsdeqlksgtasvvcllnnfypreakvqwkvdnalqsgnsqesvteqdskdstyslsstltlskadyekhkvyacevtHqgLSSPvtksfnrgec
Supplementary Figure S2. Sequence coverage of the HC and LC of mAb675 products migrating at 150 kDa and 50 kDa in non-reducing SDS-PAGE. Data derive from peptide mapping experiments on corresponding tryptic digests that were performed through MALDI-TOF-MS analysis. The identified peptides are underlined and in red. The glycopeptide (in bold) was only detected by nanoLC-ESI-Q-Orbitrap-MS/MS analysis. The leader peptides are reported in italics.
