# Supplementary material for: Production of two SARS-CoV-2 neutralizing antibodies with different potencies in Nicotiana benthamiana
Source: Front Plant Sci. 2022 Sep 5;13:956741. doi: 10.3389/fpls.2022.956741 (PMC9484322; doi:10.3389/fpls.2022.956741)
Supplement: Supplementary file 3 [file Presentation_3.PPTX]

## Slide 1
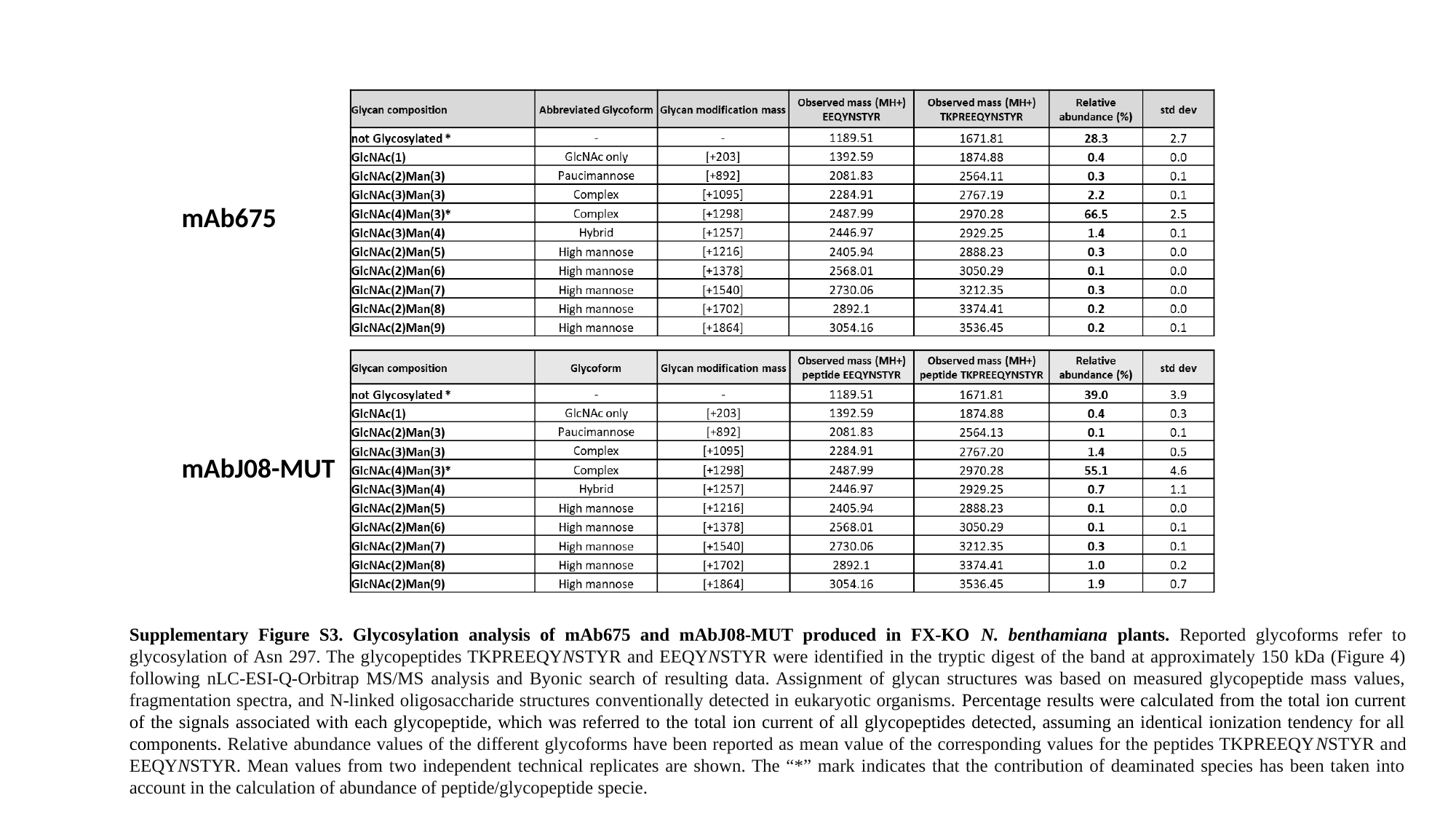

mAb675
mAbJ08-MUT
Supplementary Figure S3. Glycosylation analysis of mAb675 and mAbJ08-MUT produced in FX‐KO N. benthamiana plants. Reported glycoforms refer to glycosylation of Asn 297. The glycopeptides TKPREEQYNSTYR and EEQYNSTYR were identified in the tryptic digest of the band at approximately 150 kDa (Figure 4) following nLC-ESI-Q-Orbitrap MS/MS analysis and Byonic search of resulting data. Assignment of glycan structures was based on measured glycopeptide mass values, fragmentation spectra, and N-linked oligosaccharide structures conventionally detected in eukaryotic organisms. Percentage results were calculated from the total ion current of the signals associated with each glycopeptide, which was referred to the total ion current of all glycopeptides detected, assuming an identical ionization tendency for all components. Relative abundance values of the different glycoforms have been reported as mean value of the corresponding values for the peptides TKPREEQYNSTYR and EEQYNSTYR. Mean values from two independent technical replicates are shown. The “*” mark indicates that the contribution of deaminated species has been taken into account in the calculation of abundance of peptide/glycopeptide specie.
